# Graphite-Supported Perchloric Acid (HClO_4_-C): An Efficient and Recyclable Heterogeneous Catalyst for the One-Pot Synthesis of Amidoalkyl Naphthols

**DOI:** 10.3390/molecules18021653

**Published:** 2013-01-28

**Authors:** Zhen-Kai Lei, Li Xiao, Xiao-Quan Lu, He Huang, Chen-Jiang Liu

**Affiliations:** 1 Key Laboratory of Oil and Gas Fine Chemicals, Ministry of Education & Xinjiang Uygur Autonomous Region, Physical and Chemical Testing Center, Xinjiang University, Urumqi 830046, China; E-Mails: lzk381859468@163.com (Z.-K.L.); xiaoli56490343@163.com (L.X.); 2 Xinjiang Uygur Autonomous Region Product Quality Supervision and Inspection in Academia, Urumqi 830011, China; E-Mail: huanghe922@163.com; 3 College of Science, Beijing University of Chemical Technology, Beijing 100029, China; E-Mail: lxq1021741495@163.com

**Keywords:** amidoalkyl naphthol, solvent-free, HClO_4_-C

## Abstract

An efficient and direct protocol for the preparation of amidoalkylnaphthols employing a multi-component, one-pot condensation reaction of 2-naphthol, aromatic aldehydes and acetamide or benzamide in the presence of graphite supported perchloric acid under solvent-free conditions is described. The thermal solvent-free procedure offers advantages such as simple work-up, shorter reaction times and higher product yields, and the catalyst exhibited remarkable reactivity and can be recycled.

## 1. Introduction

Compounds bearing 1,3-amino oxygenated functional motifs are ubiquitous to a variety of biologically important natural products and potent drugs, including a number of nucleoside antibiotics and HIV protease inhibitors, such as lopinavir and ritonavir [1]. Among them, amidoalkylnaphthol derivatives are of significant and particular value because of their promising biological and pharmacological activities [2]. It has been reported that amidoalkylnaphthols can be converted to biologically active aminoalkylnaphthol derivatives by an amide hydrolysis reaction [3]. In addition, amidoalkylnaphthols can be converted to 1,3-oxazine derivatives [4]. 1,3-Oxazines have potentially different biological activities, e.g., analgesic [5] and antirheumatic properties [6], so the synthesis of amidoalkylnaphthols is of notable importance in organic synthesis.

In recent years, one-pot multicomponent reactions (MCRs) have attracted considerable attention as a result of their convergence, elegance, productivity and as powerful methods to access diverse and complex organic compounds [7,8,9]. There has been tremendous development in three- or four- component reactions. The Biginelli [10], Passerini [11], Ugi [12] and Mannich [13] reactions are some examples of MCRs, which have further led to a boom period in the study of MCRs, yet development and discovery of new MCRs is still in demand.

The synthesis of 1-amidoalkyl-2-naphthols can be carried out by multi-component condensation of 2-naphthol, aldehydes, and amides in the presence of different catalysts, such as Fe(HSO_4_)_3_ [14], LiCl [15], I_2_ [16], K_5_CoW_12_O_40_·3H_2_O [17], ionic liquids [18,19,20], MoO_3_/ZrO_2_ [21] and TrCl [22]. However, some of these catalysts suffer from the drawback of requiring the use of organic solvents, prolonged reaction times and expensive price. Therefore, the development of simple, efficient, clean, high-yielding, and environmentally friendly approaches using new catalysts for the synthesis of these compounds is an important task for organic chemists. Recently, silica-supported perchloric acid (HClO_4_-SiO_2_) has been used as a recyclable heterogeneous catalyst in this reaction [23]. Furthermore, it has been reported that graphite-supported catalysts show a certain catalytic activity in chemical reactions, such as MoO_3_-C [24], Ru and Pt-C [25], RuO_2_-C [26], Pd-C [27], LaCl_3_-C [28]. As green chemistry has become a major concern to organic chemists in present years, reactions under solvent-free conditions have received much attention [29]. With the aim of developing more efficient synthetic processes, we describe herein for the first time a new, simple, practical and inexpensive procedure for the one-pot synthesis of 1-amidoalkyl-2-naphthol derivatives via multicomponent condensation reactions between 2-naphthol, aldehydes, and acetamide or benzamide in the presence of graphite-supported perchloric acid (HClO_4_-C) as catalyst under solvent-free conditions.

## 2. Results and Discussion

**Scheme 1:**
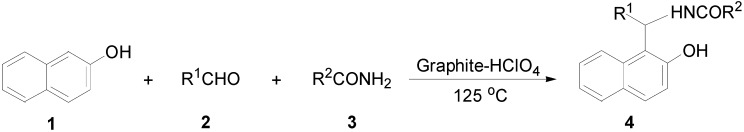
The synthesis of 1-amidoalkyl-2-naphthol derivatives catalyzed by HClO_4_-C.

Following our recent studies directed towards the development of practical, elegant and environmentally-friendly procedures for some important transformations [30,31,32], we wish to report an efficient, convenient and facile method for the condensation of 2-naphtol, aromatic aldehydes with acetamide or benzamide in the presence of graphite-supported perchloric acid as a heterogeneous catalyst leading to the corresponding amidoalkylnaphthols (Scheme 1).

To evaluate the effect of the catalyst HClO_4_-C under different reaction conditions, the reaction of 2-naphthol, benzaldehyde and acetamide was selected as a model reaction. The results are presented in Table 1. Initially, we optimized the amount of HClO_4_-C required (Table 1, entries 1–3, 5, 8) and the optimum amount was found to be 7.5 mol%. Next, the influence of the reaction time on the yield was also investigated (Table 1, entries 4–7), it was clear that the highest yield was produced when the reaction time was 2 h. Meanwhile, a slight excess of the acetamide or benzamide was found to be advantageous for yields of amidoalkylnaphthols and hence the molar ratio of 2-naphthol, aromatic aldehydes and acetamide or benzamide was kept at 1:1:1.2.

**Table 1:** Effect of the catalyst HClO_4_-C under different conditions for the reaction of 2-naphthol, benzaldehyde and acetamide ^a^.

Entry	Catalyst (mol%)	Time (h)	Yield (%) ^b^
1	1	2	68
2	2.5	2	70
3	5	2	75
4	7.5	1.5	66
5 ^c^	7.5	2	81, 80, 79, 76, 76
6	7.5	2.5	73
7	7.5	3	66
8	10	2	66

^a^ *Reaction conditions*: 2-naphthol (1 mmol), benzaldehyde (1 mmol), acetamide (1.2 mmol) and catalyst; ^b^ Isolated yields; ^c^ Catalyst was recycled five times.

The reusability of a catalyst is one of its most important benefits and makes it useful for commercial applications. Thus, when optimizing the reaction conditions, the recycling of graphite-supported perchloric acid in the reaction of 2-naphthol, benzaldehyde and acetamide was investigated. After the separation of products, the catalyst was recovered by filtration, washed with methanol (3 × 10 mL), and dried at 120 °C for 72 h. Graphite-supported perchloric acid could be recovered easily and directly reused in subsequent runs. As shown in Table 1, the desired product was obtained in 81%, 80%, 79%, 76%, 76% yields after 1–5 runs, respectively (entry 5). This indicated that the HClO_4_-C was an efficient and recyclable catalyst for the preparation of 1-amidoalkyl-2-naphthol derivatives.

In order to evaluate the generality of the process, several diversified examples illustrating the present method for the synthesis of amidoalkylnaphthols were studied (Table 2). In all cases studied, the three-component reaction proceeded smoothly to give the corresponding 1-amidoalkyl-2-naphthol derivatives in satisfactory yields. Most importantly, aromatic aldehydes with substituents bearing either electron-donating (such as methyl or methoxy) or electron-withdrawing groups (such as nitro or halide) reacted successfully in the presence of graphite-supported perchloric acid as catalyst. The use of benzamide in place of acetamide also gave similar results, as shown in Table 2 (entries 4i–o).

**Table 2:** HClO_4_-C-catalyzed one-pot synthesis of 1-amidoalkyl-2-naphthols ^a^.

Entry	R^1^	R^2^	Yield (%) ^b^	Mp (°C) ^c^	Lit. Mp (°C)
4a	C_6_H_5_	C_6_H_5_	87	238–240	238–240 [ 33]
4b	4-CH_3_C_6_H_4_	C_6_H_5_	68	218–220	214–215 [ 33]
4c	3-BrC_6_H_4_	C_6_H_5_	71	230–231	-
4d	2,4-(Cl)_2_C_6_H_3_	C_6_H_5_	96	267–269	262–263 [ 33]
4e	3-NO_2_C_6_H_4_	C_6_H_5_	83	237–239	242–243 [ 33]
4f	4-OCH_3_C_6_H_4_	C_6_H_5_	70	207–208	206–208 [ 33]
4g	4-FC_6_H_4_	C_6_H_5_	61	202–204	194–196 [ 34]
4h	3-OCH_3_-4-OH-C_6_H_3_	C_6_H_5_	84	223–225	219 [ 35]
4i	C_6_H_5_	CH_3_	81	240–242	240 [ 35]
4j	4-FC_6_H_4_	CH_3_	68	226–229	230–232 [ 14]
4k	4-CH_3_C_6_H_4_	CH_3_	74	215–217	221–223 [ 18]
4l	2,4-(Cl)_2_C_6_H_3_	CH_3_	88	207–209	202–204 [ 18]
4m	3-NO_2_C_6_H_4_	CH_3_	76	242–243	241–242 [ 18]
4n	4-ClC_6_H_4_	CH_3_	73	234–236	237–238 [ 33]
4o	3-BrC_6_H_4_	CH_3_	81	245–247	250–252 [ 34]

^a^ *Reaction conditions*: 2-naphthol (1 mmol), aldehydes (1 mmol), amide or benzamide (1.2 mmol), catalyst (0.075 mmol), solvent-free, 125 °C, 2 h; ^b^ Isolated yields; ^c^ Melting points are uncorrected.

## 3. Experimental

### 3.1. General

Melting points were determined using a Büchi B-540 instrument. All melting points are uncorrected. All compounds were characterized by IR, ^1^H-NMR spectra. The IR spectra were obtained as potassium bromide pellets with a FTS-40 spectrometer (BIO-RAD, Hercules, CA, USA). The ^1^H- and ^13^C-NMR spectra were measured on a Varian Inova-400 spectrometer (at 400 and 100 MHz, respectively) using TMS as an internal standard in CDCl_3_ or DMSO-*d_6_* Liquid aldehydes were purified by distillation prior to use.

### 3.2. General Procedure for the Preparation of HClO4-C Catalyst

70% aqueous perchloric acid (1.79 g, 12.5 mmol) was added to a suspension of graphite (4.74 g) in ether (70 mL). The mixture was concentrated and the residue was heated at 120 °C for 72 h under vacuum to give HClO_4_-C (2.64 mmol/g) as a free flowing powder (50.0 mg = 0.132 mmol of HClO_4_).

### 3.3. General Procedure for the Synthesis of Amidoalkyl Naphthols

A mixture of 2-naphthol (1 mmol), aldehyde (1 mmol), acetamide or benzamide (1.2 mmol) and HClO_4_-C (0.075 mmol) was stirred at 125 °C in oil bath and the reaction was followed by TLC. After completion of the reaction, the reaction mixture was cooled to 25 °C, then the solid residue was dissolved in boiling DMF and the mixture stirred for 5 min. The catalyst was recovered by filtration. Then solution was cooled to room temperature, the solid obtained was filtered and recrystallized from aqueous EtOH (15%). All products except **4c** are known compounds, which were characterized by mp, IR and ^1^H-NMR spectra.

*N-[(3-Bromophenyl)-(2-hydroxynaphth-1-yl)methyl)]benzamide* (**4c**): White solid. ^1^H-NMR (DMSO-*d_6_*) δ: 8.09–7.22 (m, 16H), 9.05 (d, 1H), 10.39 (s, 1H); IR: ν 3407, 3150, 1649, 1562, 1572, 1340, 1285, 817. ^13^C-NMR (DMSO-*d_6_*) δ: 49.1, 76.7, 77.0, 77.2, 77.3, 119.0, 122.8, 123.0, 123.7, 125.2, 127.2, 127.3, 128.7, 128.8 (2C), 128.9, 129.0, 129.8, 130.1, 130.2, 130.4, 132.0, 133.7, 143.5. Anal. calcd. for C_24_H_18_BrNO_2_: C, 66.68; H, 4.20; N, 3.24. Found: C, 66.60; H, 4.23; N, 3.28.

## 4. Conclusions

In summary, we have reported for the first time use of HClO_4_-C as an efficient and heterogeneous catalyst for the one-pot synthesis of a variety of amidoalkylnaphthols under solvent-free conditions. This simple procedure reported here has the advantages of mild reaction conditions, operational simplicity, catalyst showed high catalytic activity and preferable recyclability. Further work is in progress to extend the catalytic activity of HClO_4_-C to other organic transformations.
